# Identification of Microbiological Activities in Wet Flue Gas Desulfurization Systems

**DOI:** 10.3389/fmicb.2021.675628

**Published:** 2021-06-28

**Authors:** Gregory Martin, Shagun Sharma, William Ryan, Nanda K. Srinivasan, John M. Senko

**Affiliations:** ^1^Department of Biology, The University of Akron, Akron, OH, United States; ^2^Integrated Bioscience Program, The University of Akron, Akron, OH, United States; ^3^Electric Power Research Institute, Palo Alto, CA, United States; ^4^Department of Geosciences, The University of Akron, Akron, OH, United States

**Keywords:** flue gas desulfurization, thermophiles, thermoelectricity, water quality, coal combustion

## Abstract

Thermoelectric power generation from coal requires large amounts of water, much of which is used for wet flue gas desulfurization (wFGD) systems that minimize sulfur emissions, and consequently, acid rain. The microbial communities in wFGDs and throughout thermoelectric power plants can influence system performance, waste processing, and the long term stewardship of residual wastes. Any microorganisms that survive in wFGD slurries must tolerate high total dissolved solids concentrations (TDS) and temperatures (50–60°C), but the inocula for wFGDs are typically from fresh surface waters (e.g., lakes or rivers) of low TDS and temperatures, and whose activity might be limited under the physicochemically extreme conditions of the wFGD. To determine the extents of microbiological activities in wFGDs, we examined the microbial activities and communities associated with three wFGDs. O_2_ consumption rates of three wFGD slurries were optimal at 55°C, and living cells could be detected microscopically, indicating that living and active communities of organisms were present in the wFGD and could metabolize at the high temperature of the wFGD. A 16S rRNA gene-based survey revealed that the wFGD-associated microbial communities included taxa attributable to both thermophilic and mesophilic lineages. Metatranscriptomic analysis of one of the wFGDs indicated an abundance of active Burholderiaceae and several Gammaproteobacteria, and production of transcripts associated with carbohydrate metabolism, osmotic stress response, as well as phage, prophages, and transposable elements. These results illustrate that microbial activities can be sustained in physicochemically extreme wFGDs, and these activities may influence the performance and environmental impacts of thermoelectric power plants.

## Introduction

Industrialized nations rely heavily on coal-fired thermoelectric power plants to support energy needs. In the United States, 24% of electricity is furnished by coal burning power plants (U.S. Energy Information Agency (USEIA), 2020). Coal combustion remains one of the largest contributors of air pollution including Hg, and acid rain-forming NO_*x*_ and SO_2_ (Likens et al., 1996; Srivastava and Jozewicz, 2001). Flue gas (coal exhaust) desulfurization units (FGDs), intended to remove SO_2_, NO_*x*_, and, to a lesser extent, Hg^2+^, were introduced starting in the 1970s to mitigate the acid rain problem (Srivastava et al., 2006; Córdoba, 2015). In wet FGDs (wFGDs), flue gas is passed into a tank above a limestone or slaked lime slurry, and wetted by spray nozzles, whereby SO_2_ partitions into the slurry. Ca^2+^ in the limestone or lime then forms a CaSO_3_ precipitate slurry, which can then be further oxidized to gypsum (CaSO_4_.2H_2_O), with the addition of O_2_ (also called forced oxidation) (Córdoba, 2015). The scrubbed gas can continue through the top of the system and exits the stack to the atmosphere. The bottom slurry mixture is cycled out of the tank for processing. The solids are dewatered and landfilled or sold for industrial uses such as wallboard, cement, and soil augmentation to offset operational and disposal costs. The liquor, which contains high dissolved solids, and a variety of potentially toxic chemical (e.g., Hg, As, and Se) is either recycled into the wFGD system, or purged for halogen removal and treated before discharge to a large water body.

Microorganisms have been observed in wFGDs, but the high temperatures (50–60°C) induced by the exhaust, and high concentrations of Ca^2+^, Mg^2+^, SO_4_^2–^, Cl^–^, and toxic metals make wFGDs harsh environments for microbial growth (Brown et al., 2012; Córdoba, 2015). The microbial communities and activities associated with wFGDs remain poorly understood despite their importance in FGD performance and downstream processing of waste streams (Buchart et al., 2006; Córdoba, 2015; Graves et al., 2017; Gingerich et al., 2018). Most notably, microbial activities in wFGDs have been implicated in the corrosion of unit-associated structures and the “re-emission” of Hg (Barkay and Wagner-Döbler, 2005; Wu et al., 2010; Moskal, 2011; Moretti and Jones, 2012; Ochoa-González et al., 2013). In the latter process, Hg, which would otherwise be retained in the wFGD as Hg^2+^ adsorbed to solids in the slurry is reduced to volatile Hg^0^, potentially by microbiological activity, and emitted into the atmosphere (Barkay and Wagner-Döbler, 2005; Wu et al., 2010; Moretti and Jones, 2012; Ochoa-González et al., 2013). Despite speculation of the activities of micoorganisms in wFGDs, it remains unclear if they are, in fact, active.

Given the relatively harsh physicochemical conditions associated with the interior of wFGDs (i.e., high temperatures and dissolved solids concentrations), it is reasonable to expect that microbial abundances and activities associated with these systems would be minimal. Indeed, diversity of microbial communities associated with wFGD slurries is lower compared to those associated with waters that did not experience the relatively harsh conditions of the wFGD interior (Brown et al., 2012). Several classes of organisms detected in wFGDs were similar to those detected in waters used in slurry preparation (lake or river water), indicating that many of the organisms in wFGD slurries were present, but not necessarily active. However, the potential for microbial activity in wFGDs clearly exists. For instance, microorganisms capable of growth in high-sulfate medium at 60°C were shown to be more abundant in wFGD slurries than in fresh waters used to fills the wFGDs (Brown et al., 2012). Microbial communities associated with the deposits on the interior walls of wFGDs contained classes of organisms encountered in geothermal settings (Brown et al., 2012). Some of these observations could indicate that microbial activity is minimal in wFGDs, but they also indicate that residence times of fluids in the slurries might be such that the physicochemical conditions of the systems exert some control on the structure of the microbial communities. They also indicate that while the inoculum for wFGDs is derived from less physicochemically extreme ecosystems (i.e., river or lake water and limestone), the relatively harsh conditions of the wFGD interior select for unique, active microbial communities in wFGDs. In summary, past work indicates that microorganisms were present, and some were culturable in wFGDs (Brown et al., 2012), but whether they were active, and the extents of their activities remain unclear. Therefore, the goal of this work was to understand the composition and activities of microorganisms associated with wFGDs, by (1) establishing how organisms in wFGDs differ from the freshwater sources of slurry water, and (2) determining if organisms in wFGDs are alive and actively metabolizing.

## Materials and Methods

### Sampling Sites and Collection

In August 2016, samples were collected from three coal-fired thermoelectric power plants on the Ohio River, all of which are within a 210 km stretch of the river, and operate multiple wFGDs with the river as their source water. The temperature of all the wFGDs is maintained near 55°C. The first site, S, relies on limestone wFGD with forced air oxidation, the second site, M, uses a magnesium lime mixture in its wFGDs, and the third site, P, operates a modified magnesium lime system with higher MgOH levels. Thirty 50 mL vials and two 1 L bottles of slurry were collected from bleed valves adjacent to wFGDs of sites S, M, and P. Two 1 L bottles of river water were collected near the system intake at sites S and P. All samples were collected in sterile containers and transported on ice to The University of Akron (UA) for further analysis. Immediately upon return to the laboratory, for each site, 5 mL of sample was filter sterilized into 5 mL of 0.5 M HCl for atomic absorption spectrometry analysis, 5 mL of sample was filter sterilized in preparation for anion quantification (described below), and samples intended for DNA extraction were stored in a −80°C freezer, all other samples were placed in the refrigerator. A 20 mL sample from Unit P that was intended for metatranscriptomic analysis was suspended in 20 mL DNA/RNA Shield (Zymo Research, Irvine, CA, United States) RNA stabilization solution before transport on ice to UA (≤2 h), where it was then stored at −80°C before further processing.

### wFGD Slurry Incubations

Aerobic microbial activities in slurries were determined by measuring O_2_ consumption rates using a Micro-Oxymax respirometer (Columbus Instruments, Columbus, OH, United States) (Poncelet et al., 2013). Temperatures were maintained in a water bath and microbiological activities were deactivated by autoclaving wFGD slurry for negative controls. Slurry incubations were carried out at 20, 40, 55, and 70°C in triplicate for non-sterile incubations and duplicate for deactivated ones.

### Microbial Enumerations and Enrichments

Microbial cell abundances in wFGD slurries were determined by total live/dead cell counts after staining samples with SYTO 9 green-fluorescent nucleic acid stain and propidium iodide red-fluorescent nucleic acid stain (Thermo Scientific Inc., Waltham, MA, United States), followed by visualization and cell counting on an Olympus BX53F fluorescent light microscope (Olympus Life Science Solutions, Waltham, MA, United States). Briefly, 0.5 mL of each sample was stained according to the *BAC*Light protocol and then transferred into wells on TEKON black Teflon coated 12 well slides (TEKON Inc., Myakka City, FL, United States). Five random grids from five wells were counted and then averaged per sample, for both live and dead stains. Only cells that were brightly stained and clearly differentiated from the background florescence were included in the counts. A test case was also conducted, where live *Shewanella oneidensis* cells were added to slurry in a 1:1 ratio, and stained to ensure that cells could be differentiated from slurry solid phases (Supplementary Figure 1). Cell counts per field are shown in Supplementary Table 1.

Aerobic enrichments from wFGD slurries were carried out with culture medium (pH 6.5) that contained CaCO_3_ (3 g/L), NH_4_Cl (0.16 g/L), K_2_HPO_4_ (0.09g/L), NaH_2_PO_4_ (0.06 g/L), MgBr_2_⋅6H_2_0 (0.09 g/L), MgSO_4_⋅7H_2_0 (17 g/L), and MgCl_2_⋅6H_2_0 (6 g/L), vitamins and trace metals (Tanner, 1997), and glucose (0.9 g/L) or acetate (1.64 g/L) as electron donors and carbon sources. Enrichments were incubated at 50°C, and maintained through six transfers before they were harvested and characterized using 16S rRNA gene sequence surveys.

### Nucleic Acid-Based Microbial Community Characterization

Before nucleic acid extraction for 16S rRNA gene surveys, suspended solids from the wFGD slurries or enrichment cultures were concentrated by centrifugation at 3000 × *g* for 10 min and planktonic organisms in Ohio River wFGD source water were concentrated by filtration (0.2 μm). Pellets and filters were stored at −70°C until DNA was extracted using MoBio PowerBiofilm DNA isolation kits (MoBio Laboratories, Inc., Carlsbad, CA, United States), and DNA was quantified using a Qubit 3.0 Fluorometer (Thermo Scientific Inc., Waltham, MA, United States). DNA from all wFGDs and source water samples was shipped to Molecular Research LP (MR DNA, Shallowater, TX, United States) for Illumina MiSeq sequencing (Illumina, Inc., San Diego, CA, United States), where barcoded 515F, 806R primers (Caporaso et al., 2011) were used to amplify partial 16S rRNA genes through a 28 cycle PCR using the HotStarTaq Plus Master Mix Kit (Qiagen, United States) programed for 94°C for 3 min, then 28 cycles of 94°C for 30 s, 53°C for 40 s, and 72°C for 1 min, and 72°C for 5 min final elongation. Multiple samples were pooled in equal proportions based on their molecular weight and DNA concentrations, as determined by a 2% agarose gel. The Illumina DNA library was prepared from calibrated AMpure XP bead purified pooled samples. Amplicons were sequenced on an Illumina MiSeq, following the manufacturer’s guidelines, and then barcodes were depleted, <150 bp sequences were removed, sequences with ambiguous base calls were removed, and the sequences were denoised and chimeras removed using the quality filtering strategy described by Bokulich et al. (2013).

16S rRNA gene sequences were processed using default parameters of QIIME 1 scripts (Caporaso et al., 2010). Operational taxonomic units based on 97% sequence similarity (OTU_0_._03_) were picked *de novo* and assigned to taxonomic units using the RDP Classifier 2.2 with the SILVA database (Wang et al., 2007; Edgar, 2010; Werner et al., 2012). 86,851–135,615 sequences were obtained from the slurries or river water samples, and within them, 1,665–4,123 unique OTUs were identified (Supplementary Table 2). wFGD sequence libraries from each sample were rarefied to 24,439 sequences, and Shannon diversity indices were calculated (Lozupone and Knight, 2005; Price et al., 2010; Caporaso et al., 2011; Quast et al., 2013). Abundant OTU_0_._03_ in libraries were compared to sequences in the National Center for Biotechnology Information (NCBI) database using the Basic Local Alignment Search Tool (BLASTN; Altschul et al., 1997).

For metatranscriptomic analysis of Unit P microorganisms, slurry sample was shipped on dry ice to MR DNA Lab for total RNA sequencing. Slurry solids were concentrated by centrifugation and total RNA was extracted from the pellet using the MoBio RNA PowerSoil Total RNA Isolation Kit (MoBio Laboratories, Inc., Carlsbad, CA, United States). DNA contamination was removed using Baseline-ZERO DNase (Lucigen Corporation, Middleton, WI, United States) and samples were purified using RNA Clean and Concentrator Columns (Zymo Research, Irvine, CA, United States). Next, whole transcriptome amplification was conducted using QuantiTect Whole Transcriptome Kit (Qiagen, Germantown, MD, United States) and Nextera DNA Sample preparation. Double stranded cDNA concentrations were measured with a Qubit 3.0 Fluorometer (Thermo Scientific Inc., Waltham, MA, United States), and the diluted samples were fragmented, and adapter sequences and unique indices were added during a 5 cycle PCR. Final library concentrations were measured with a Qubit 3.0 Fluorometer (Thermo Scientific Inc., Waltham, MA, United States) and average library size was determined with an Agilent 2100 Bioanalyzer (Agilent Technologies, Santa Clara, CA, United States). Libraries were pooled in equimolar ratios of 2 nM. 10 pM of the pooled library was clustered using Illumina cBot and paired end sequenced for 500 cycles on an Illumina HiSeq 2500 system.

Unpaired sequence reads were processed using MetaGenomics Rapid Annotation using Subsystem Technology (MG-RAST) pipeline version 4.0^1^ (Meyer et al., 2008). Quality control included removal of sequences with greater than 10 ambiguous bases per read and dereplication of duplicate sequences with identical sequences in the 50 bp. After quality control, a total of 695 Mbp were recovered, which included 4.1 × 10^6^ sequences of mean length 170 bp. Taxonomy of 16S rRNA was assigned based on the M5nr database (Wilke et al., 2012) using BLASTX in MG-RAST with an *e*-value of 10^–5^ and minimum identity of 60%, revealing 679 predicted rRNA features. Functional assignments were made to other reads based on the SEED database (Overbeek et al., 2005) using BLASTX in MG-RAST with an *e*-value of 10^–5^, revealing 74,406 predicted protein features. Supplementary Table 3 provides metatranscriptomic metadata. Both the Unit P metatranscriptome and partial 16S rRNA gene sequences reported in this paper are available in the Sequence Read Archive (SRA) under BioProject accession number PRJNA689629.

### Analytical Techniques

Cl^–^, NO_3_^–^, and SO_4_^2–^ concentrations were measured with a Dionex ICS-1100 Basic Integrated IC System (Thermo Fisher Scientific Inc., Sunnyvale, CA, United States). Dissolved Ca and Mg concentrations were measured with a Perkin Elmer AAnalyst 700 Atomic Absorption Spectrometer (PerkinElmer Corp., Norwalk, CT, United States). pH was measured with a VWR^®^ SympHony^TM^ B10P Benchtop Meter (VWR Int., Radnor, PA, United States). Glucose concentrations were determined using colorimetric method with a glucose assay kit (STA-680, Cell Biolabs, Inc., San Diego, CA, United States) on a spectrophotometer (Spectra max 384 plus; Molecular devices, Sunnyvale, CA, United States) at 540 nm. Changes in the acetate concentrations in the medium were quantified by high-performance liquid chromatography, using a Shimadzu LC-10A HPLC system (Shimadzu Scientific Instruments, Inc., Columbia, MD, United States) with Aminex HPX-87H column (300 mm, 7.8 mm; Bio-Rad Laboratories, Inc., Hercules, CA, United States) with 0.008 M H_2_SO_4_ as a mobile phase, a flow rate of 0.6 ml/min, and UV (254 nm) detection (SPD-10A).

## Results and Discussion

### wFGD Slurry Chemistry

Samples were collected from three wFGDs of coal-fired thermoelectric power plants, all located in the Ohio River watershed, and designated Units S, M, and P. Unit S is a limestone-based wFGD that uses forced air to oxidize calcium sulfite to calcium sulfate (gypsum), and Units M and P use lime as a Ca source for SO_*x*_ precipitation and are often enhanced with MgOH for higher alkalinity. Both types of reactors are characterized by high dissolved solids concentrations, which vary depending on the residence times of solids and liquid phases, coal type, power generation intensity, and operator preferences (Córdoba, 2015). At the time of sample collection, all units contained high sulfate concentrations with slightly acidic pH (Table 1). While the chloride concentration was low in Unit S, it was high in Units M and P, likely due to differences in operation, residence time, or coal type (Córdoba, 2015). Despite these differences, at the relatively high operating temperatures (approximately 55°C) and dissolved solids concentrations, these systems represent a pysicochemical extreme environment in comparison to the freshwater (i.e., lake or river) sources of water that are used to fill the reactors (Table 1). While pH was the only parameter of river water chemistry measured in this work, chloride and sulfate concentrations are typically approximately 0.8 and 0.7 mM, respectively, in the large rivers of the Ohio River watershed on which the power plants are located (Ohio River Valley Water Sanitary Commission (ORANCO), 2018). The major anions in the wFGDs are considerably higher than those encountered in freshwater systems (Table 1). However, the wFGDs would not classified as saline environments because that term is generally applied to dissolved NaCl (e.g., Merino et al., 2019). In the wFGDs, chloride concentrations vary, but sulfate, magnesium, and calcium concentrations are 4–5 times higher than would be encountered in seawater (Table 1).

**TABLE 1:** Aqueous chemistry, microbial cell abundances, and non-parametric microbial community diversity indicators of wFGD slurry and source water. All measurements were made on samples collected in August, 2016, except for cations, which were measured in samples collected in August 2015. n/d = not determined. Ocean water chemistry is included for comparison to the wFGDs.

Sample	pH	Cl^–^ (mM)	NO_3_^–^ (mM)	SO_4_^2–^ (mM)	Mg^2+^ (mM)	Ca^2+^ (mM)	Live cell abundance (cell/mL)	Dead cell abundance (cell/mL)	Shannon index
Unit S	5.44	0.0	4.6	124	163	104	1.8 × 10^6^	2.7 × 10^6^	6.02
Unit M	6.87	98	0.0	193	167	45	7.5 × 10^6^	5.4 × 10^6^	4.17
Unit P	5.82	110	0.0	205	402	44	4.4 × 10^7^	4.9 × 10^6^	5.34
S Source^1^	7.73	0.8	0.01	0.7	0.4	0.8	n/d	n/d	6.80
P Source^1^	7.75	0.8	0.01	0.7	0.4	0.8	n/d	n/d	6.08
Ocean water^2^	7.84	550	0.0	28	53	10			

*^1^All values except pH are from Ohio River Valley Water Sanitary Commission (ORANCO) (2018).*

*^2^Values are from Turekian (1968).*

### Microbial Activity in wFGD

**FIGURE 1:**
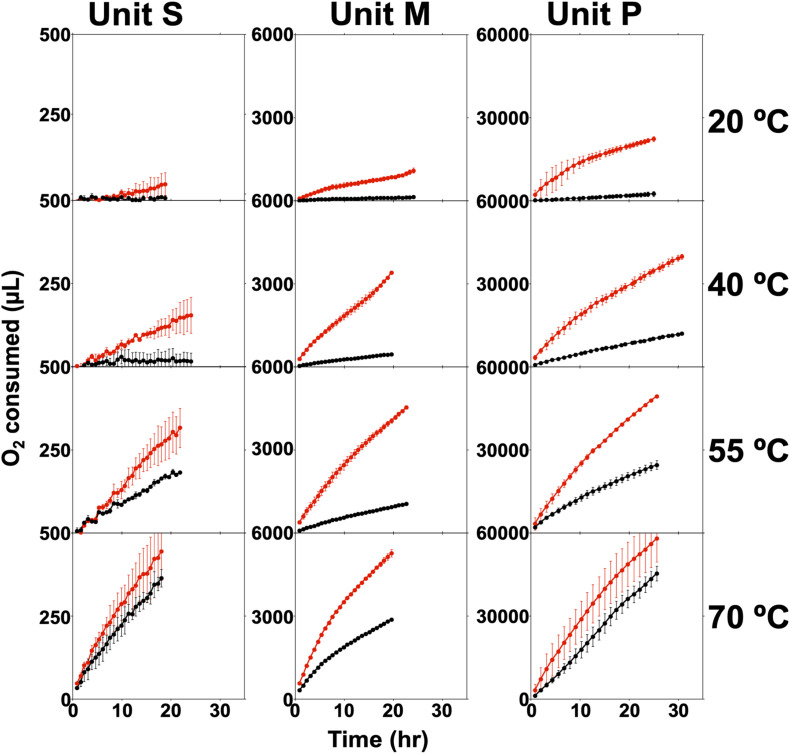
O_2_ consumption in non-sterile (red) and autoclaved (black) wFGD slurry material from Unit S, Unit M, and Unit P incubated at 20, 40, 55, and 70°C. Error bars represent one standard deviation of triplicate (non-sterile) or duplicate (autoclaved) incubations.

During wFGD startup, water from a nearby source is used for the slurry, and this water is also used to makeup water lost during operation (Ochoa-González et al., 2013; Córdoba, 2015). Therefore, microorganisms associated with relatively low-temperature, fresh water sources are exposed to high temperatures and dissolved solids concentrations in the wFGD (Brown et al., 2012). Such harsh conditions could limit activity of the mostly mesophilic source water-associated microorganisms once in the wFGD. Previously, some evidence of microbial activity was suggested by adipic acid consumption in wFGD slurries (Buchart et al., 2006). Live/dead staining and fluorescence microscopy were used to assess whether cells in all three wFGDs were alive. Units S and M contained approximately 1:1 live:dead (Table 1), with the fewest total cells observed in Unit S (Table 1). Unit P contained the most microbial biomass, and approximately 90% of the cells were alive (Table 1). As an indicator of microbiological activity, O_2_ consumption by non-sterile wFGD slurry exceeded that of the autoclaved slurry in all of the incubations, except when Unit S slurry was incubated at 70°C (Figure 1). Generally, abiotic O_2_ consumption (i.e., by autoclaved wFGD slurry) increased with increasing temperature, but the difference between abiotic and biological O_2_ consumption was greatest at 40 and 55°C in all cases. These results indicate that the microbial communities associated with the slurries metabolize optimally at wFGD operating temperatures, but can also metabolize at lower temperatures. The microbial communities appear to have changed in response to the physical conditions of the wFGDs and metabolize optimally at the relatively high temperatures of these system. Similarly, O_2_ consumption rates corelated with live cell abundances, with Unit S exhibiting the least activity, and Unit P exhibiting the greatest activity (Table 1 and Figure 1).

### Microbial Community Composition

**FIGURE 2:**
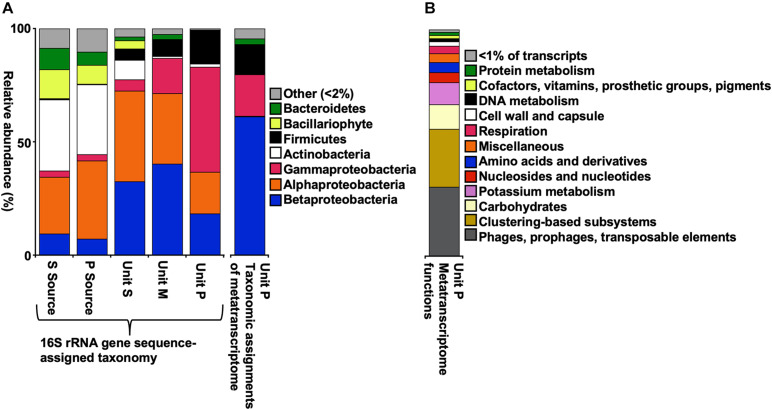
Relative abundances of 16S rRNA gene sequencing-derived OTU at the phylum-level (class-level for Proteobacteria) in wFGD source waters and units, consensus taxonomic assignments of 16S rRNA in the Unit P transcriptome **(A)**, and functional assignments of transcripts detected in Unit P **(B)**.

Source waters represent a relatively low-temperature and dissolved solids concentration inoculum for the wFGDs (Brown et al., 2012). Source water for both Units P and S contained abundant Actinobacteria, Alphaproteobacteria, and Betaproteobacteria, with composition similar to other observations of the Ohio River watershed (e.g., Schultz et al., 2013; Stannish et al., 2016). In both river libraries, Actinobacteria were predominantly the aquatic HGCI (Warnecke et al., 2004) and CL500-29 (Xu et al., 2018). Alphaproteobacteria were predominantly the widely distributed uncultured SAR11 freshwater clade LD12 (Salcher et al., 2011), and Betaproteobacteria were predominantly the freshwater planktonic *Polynucleobacter* (Hahn et al., 2016). Taken together, the source water-associated microbial communities were typical of freshwater systems. The Shannon diversity indices of wFGDs were slightly lower than those of the source water (Table 1), and the wFGDs exhibited different phylum-level community composition patterns than the source water (Figure 2A). Both of these differences were likely induced by the relatively harsh physicochemical conditions of the wFGDs. Archaea- and Eukarya-assignable sequences comprised ≤0.05% of the sequences in our libraries. While all three wFGD slurries contained abundant phylotypes attributable to Alphaproteobacteria and Betaproteobacteria (Figure 2A), genus-level taxonomic assignments within these phyla varied among the three units. *Albidovulum-* (Alphaproteobacteria), *Gallionella*-, *Thiomonas*-, and *Hydrogenophaga*- (Betaproteobacteria) affiliated phylotypes were abundant in Unit S, *Rhodobacter*- (Alphaproteobacteria) and *Hydrogenophilus*- (Betaproteobacteria) affiliated phylotypes were abundant in Unit M, and *Sphingomonas*- (Alphaproteobacteria), *Ralstonia*-, and *Tepidimonas*- (Betaproteobacteria) affiliated phylotypes were abundant in Unit P. *Pseudomonas*- (Gammaproteobacteria), and *Atopostipes*- (Firmicutes) affiliated phylotypes were also abundant in Unit P. The most prominent taxa in Units S and M were mostly lithotrophic and/or thermophilic (*Albidovulum*, *Gallionella*, *Thiomonas*, *Hydrogenophaga*, *Rhodobacter*, and *Hydrogenophilus*; Willems et al., 1989; Hallbeck and Pedersen, 1991; Hayashi et al., 1999; Albuquerque et al., 2002; Ghosh and Dam, 2009; Arsène-Ploetz et al., 2010), while those in Unit P were mostly organotrophic mesophiles (except for Tepidomonas; Schönfeld et al., 2003; Cotta et al., 2004; Leys et al., 2004; Chen et al., 2013; Al-Mailem et al., 2019), which would not be expected to be active. Nevertheless, Unit P exhibited the most abundant live cells and O_2_ consumption activity (Table 1 and Figure 1). At the phylum level, community composition of the three wFGDs studied here, were similar to those observed by Brown et al. (2012), with Alphaproteobacteria, Betaproteobacteria, Gammaproteobacteria, and Actinobacteria most abundant. Additionally, genera including *Hydrogenophilus*, *Ralstonia*, and *Pseudomonas* were encountered in the current units as well as in previously characterized wFGD slurries (Brown et al., 2012).

### Identification of Active Organisms in Unit P

**FIGURE 3:**
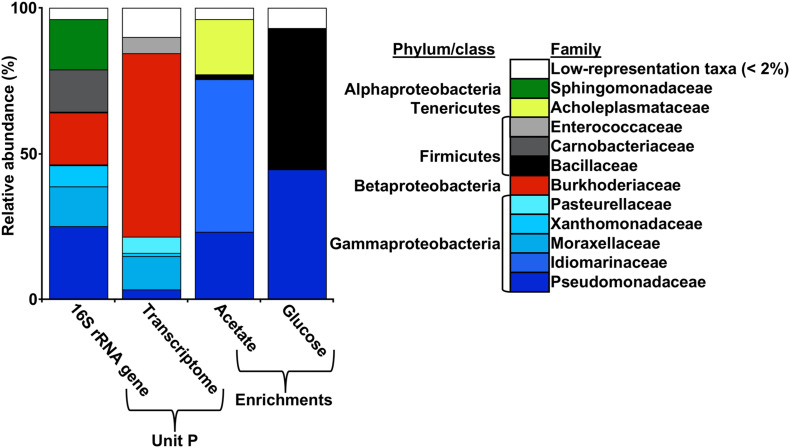
Relative abundances of 16S rRNA gene sequencing-derived OTU at the family level, consensus family level taxonomic assignments of 16S rRNA in the Unit P transcriptome, and 16S rRNA gene sequencing-derived family level taxonomic composition of aerobic acetate- and glucose-oxidizing enrichment cultures from Unit P.

In previous work, wFGD microbial communities were found to consist of a mixture of thermophilic (e.g., *Hydrogenophilus*) and mesophilic (e.g., *Pseudomonas*) taxa (Brown et al., 2012). These observations led to the conclusion that the mesophilic taxa were inactive remnants of the community associated with the freshwater source, while the thermophilic taxa were enriched in the harsh physicochemical setting of the wFGD interior. To identify the active organisms in the wFGD setting, 16S rRNA gene sequences in the metatranscriptome from the Unit P slurry was compared to those in the 16S rRNA gene survey of the Unit P microbial community (Figure 2A). Taxonomic assignment of 16S rRNA in the transcriptome revealed that the most abundant active phyla in Unit P slurry were Firmicutes, Gammaproteobacteria, and Betaproteobacteria (Figure 2A). The Firmicutes-attributable transcripts were assigned to the Enterococcaceae, which have been cultivated from a thermophilic biogas production facility (Maus et al., 2016). The Betaproteobacteria-attributable transcripts were assigned with further taxonomic resolution to the Burkholderiaceae, including *Ralstonia/Cupriavidus* (Figure 3), of which thermophilic representatives have been isolated (Vandamme and Coenye, 2004; Sheu et al., 2012; Shah et al., 2015). While active Betaproteobacteria were attributable to potentially thermophilic lineages, this was not the case with the Gammaproteobacteria, among which, transcripts attributable to Moraxellaceae, Pasteurellaceae, Pseudomonadaceae were abundant (Figure 3). These included *Psychrobacter*, *Histophilus*, and *Pseudomonas*. Neither *Psychrobacter* nor *Histophilus* contain thermophilic representatives. *Psychrobacter* are well-known phsychrophiles (Bendia et al., 2018), and *Histophilus* are obligately parasitic (Hansen et al., 2013). While *Pseudomonas* spp. are cosmopolitan and observed in non-extreme settings, thermophilic and thermotolerant representatives have been described (Hollocher and Kristjánsson, 1992; Manania and Moore, 2002). Indeed, they are observed among other mesophiles in thermal environments (Droffner et al., 1995), indicating that classifications of organisms typically described as mesophiles are capable of survival and growth at temperatures well outside their described range. In previous work relying only on 16S rRNA gene surveys, it was presumed that these predominantly mesophilic lineages (including *Pseudomonas*) were present, but inactive components of wFGD microbial communities (Brown et al., 2012), but that does not appear to be the case.

Besides metatranscriptomic evidence of active organisms in Unit P slurry, thermophilic, aerobic, acetate and glucose oxidizing enrichment cultures were recovered in media designed to mimic wFGD chemistry. Growth was assessed based on direct cell counts and glucose or acetate depletion (Supplementary Figure 2). After six transfers in these media and incubation at 55°C, we detected phylotypes attributable to Pseudomonadaceae (Figure 3). A comparison of the Pseudomonadaceae-affiliated OTU to 16S rRNA gene sequences in the GenBank database using BLASTn (Altschul et al., 1997) revealed that it was most closely related (99% identity) to a *Pseudomonas mendocina* strain CB46 from hydrocarbon-contaminated soil (Al-Mailem et al., 2019). Pseudomonads were prominent components of the Unit P microbial community based on the 16S rRNA gene survey, in the metatranscriptome-based community profile, and in enrichment cultures (Figure 3), indicating that these organisms are important and active parts of the microbial community in Unit P. Additionally, organisms attributable to marine, aerobic, organotrophic *Idiomarina* sp. (Sanz-Sáez et al., 2019), a marine Tenericutes (Fichtel et al., 2012), and a *Bacillus subterraneus* from Cr(VI)-contaminated soil (Liu et al., 2019) were abundant in enrichment cultures. However, these latter organisms were not prominent components of the Unit P microbial community as indicated by the 16S rRNA gene sequencing-based survey or taxonomic assignment of 16S rRNA in the metatranscriptome (Figure 3).

An analysis of the functions of gene products detected in the Unit P metatranscriptome provided an indication of the microbial response to the harsh physicochemical conditions of the wFGD interior. Most of the genes detected were attributable to potassium metabolism, carbohydrate metabolism, clustering-based subsystems, and phages and transposable elements (Figure 2B). Clustering-based subsystems are genetic systems with unknown or unclear predicted functions (Overbeek et al., 2005). The relatively high expression of genes involved in potassium metabolism are likely a response to the osmotic stress associated with the wFGD, which are hypertonic solutions, containing notably high concentrations of chloride, sulfate, magnesium, and calcium (Table 1). Under these conditions, cells may transport K^+^ into the cytoplasm to minimize this osmotic stress imposed by wFGD fluids (Rosen, 1986; Booth and Higgins, 1990; Meury and Kohiyama, 1992; Guitierrez et al., 1995). Genes SEED database (Overbeek et al., 2005) classified in the potassium metabolism subsystem include the *ktr* potassium uptake systems, which are involved in short- and long-term osmotic stress response (Holtman et al., 2003).

Transcription of genes involved in carbohydrate metabolism, as well as amino acid metabolism (Figure 2B), may have been a response to osmotic stress (e.g., synthesis of osmoprotectants; Booth and Higgins, 1990; Guitierrez et al., 1995; Poolman and Glaasker, 1998). However, carbohydrate metabolism was also likely devoted to energy recovery, as previous work has shown that wFGD slurries contain abundant dissolved organic carbon (mostly from fly ash; Brown et al., 2012), and this endogenous organic carbon was likely the major driver of O_2_ consumption in the respirometry incubations (Figure 1). The prevalence of phage-attributable signals in the transcriptome could be due to “kill the winner” patterns of phage reproduction, whereby relatively rapidly growing organisms are most susceptible to infection (Parmar et al., 2018). Phage appear to be more prominent in microbial communities that are undergoing relatively dramatic physicochemical perturbation and in cases where microbial activity is enhanced (Pan et al., 2014; Trubl et al., 2018). Indeed, a metagenomic survey of microbial communities associated with hydraulic fracturing return waters revealed that phage were increasingly prominent when microbial communities from freshwater-based fracking fluid were incubated in high-temperature, hypertonic shale plays (Daly et al., 2019), which could be considered physicochemical analogs of wFGDs.

### Impacts of Microbial Activities on Thermoelectric Power Plant Performance

Thermoelectric power generation (whether coal, natural gas, or nuclear) accounts for approximately 40% of water withdrawal in the United States, with approximately 500 million cubic meters of water used per day in 2015 (Dieter et al., 2018). This water is used for steam generation, cooling, and waste product capture, transport, and disposal (Yang and Dziegielewski, 2007; Badr et al., 2012; Dieter et al., 2018). In many ways, the water used in electric power generation, represents the circulatory system of the electric power plant, because it facilitates the transfer of energy and material necessary for plant operation. As it circulates, the water and the microorganisms associated with it are exposed to dramatically differing physicochemical conditions. These fluctuating conditions exert control on the microbiological activities which can then influence system performance and environmental impacts of electric power generation. The work here shows some of these shifts.

Despite the importance of freshwater use in thermoelectric power generation the activities of microorganisms in these systems have been mostly unexplored, though invoked as causes of operational problems. A goal of this paper was to establish the composition and activity of microbial communities in wFGDs, and the establishment of activity sets the stage to explore how they influence the performance of thermoelectric power facility performance. For instance, microbiological activities have been suggested to be responsible for excessive foaming in wFGDs and corrosion of steel structures of power plants (Rajendran et al., 1999; Buchart et al., 2006; Moskal, 2011; Brown et al., 2012; Qin et al., 2013, 2014). In the wFGD, microbial activities might be used to enhance the removal of SO_2_ and gypsum formation (Huber and Stetter, 1998; Zhang and Liu, 2015; Graves et al., 2017; Zhang et al., 2017), while also modulating the redox state of Hg that passes through the wFGD unit, where Hg^0^ oxidation could limit its release and Hg^2+^ reduction could enhance its release into the atmosphere (Barkay and Wagner-Döbler, 2005; Wu et al., 2010; Moretti and Jones, 2012; Ochoa-González et al., 2013). Finally, microbial activities can influence the fate of the most prominent toxic components of solid waste, As, Se, Pb, and Hg (Gingerich and Mauter, 2020), as well as enhance the removal of toxic dissolved species such as Se (Van Ginkel et al., 2011; Nakajima et al., 2013; Andalib et al., 2016; Córdoba and Staicu, 2018; Gingerich et al., 2018). In all of these scenarios, microbial communities might be subjected to changes in their physicochemical setting as water and suspended solids move through thermoelectric power generation station process points, thus forcing shifts in microbial communities, and potentially, activities. From the perspective of the microorganisms detected in the wFGDs in the current work, the abundant *Hydrogenophilus, Hydrogenophaga*, and *Gallionella*, which were abundant in Units S and M, could induce corrosion (Rajala et al., 2015; Huang et al., 2021). Additionally, active organisms in Unit P, including *Ralstonia*/*Cupriavidus* spp. and *Pseudomonas* spp. have been shown to induce steel corrosion as well as Hg(II) reduction, which could lead to Hg reemission (Da Silva et al., 2002; Champier et al., 2004; Pepi et al., 2011; Rojas et al., 2011; Li et al., 2016; Jia et al., 2017; Zheng et al., 2018). Organisms within these taxa can also mediate environmentally beneficial processes, such as the reductive immobilization of Se (Roux et al., 2001; Hunter, 2014).

It is also important to note that the same large river system provided the source water for all three of the wFGD units examined here, but despite the similarities in source water-associated microbial communities (Figure 2A), distinct patterns of microbiological community composition and activity were observed in the wFGDs. This observation suggests that power generation station-specific operational characteristics might be responsible for how microbial communities are shaped within different process points of the facilities. Despite the differences among the units, this work shows that a prominent subset of the microbial community in a physicochemically extreme human-made setting is actively metabolizing, and therefore, could influence the performance of that system.

## Data Availability Statement

The datasets presented in this study can be found in online repositories. The names of the repository/repositories and accession number(s) can be found below: https://www.ncbi.nlm.nih.gov/, PRJNA689629.

## Author Contributions

GM: conceptualization, data curation, formal analysis, investigation, methodology, validation, visualization, writing – original draft, and writing – review and editing. SS: formal analysis, investigation, methodology, supervision, and writing – original draft. WR: investigation, methodology, and writing – original draft. NS: methodology, project administration, and writing – original draft. JS: conceptualization, data curation, formal analysis, funding acquisition, investigation, methodology, project administration, resources, supervision, writing – original draft, and writing – review and editing. All authors contributed to the article and approved the submitted version.

## Conflict of Interest

The authors declare that the research was conducted in the absence of any commercial or financial relationships that could be construed as a potential conflict of interest.

## Footnotes

1 https://www.mg-rast.org
