# Supplementary material for: Identification of Microbiological Activities in Wet Flue Gas Desulfurization Systems
Source: Front Microbiol. 2021 Jun 28;12:675628. doi: 10.3389/fmicb.2021.675628 (PMC8273512; doi:10.3389/fmicb.2021.675628)
Supplement: Supplementary file 1 [file Data_Sheet_1.docx]

**Supplemental information**


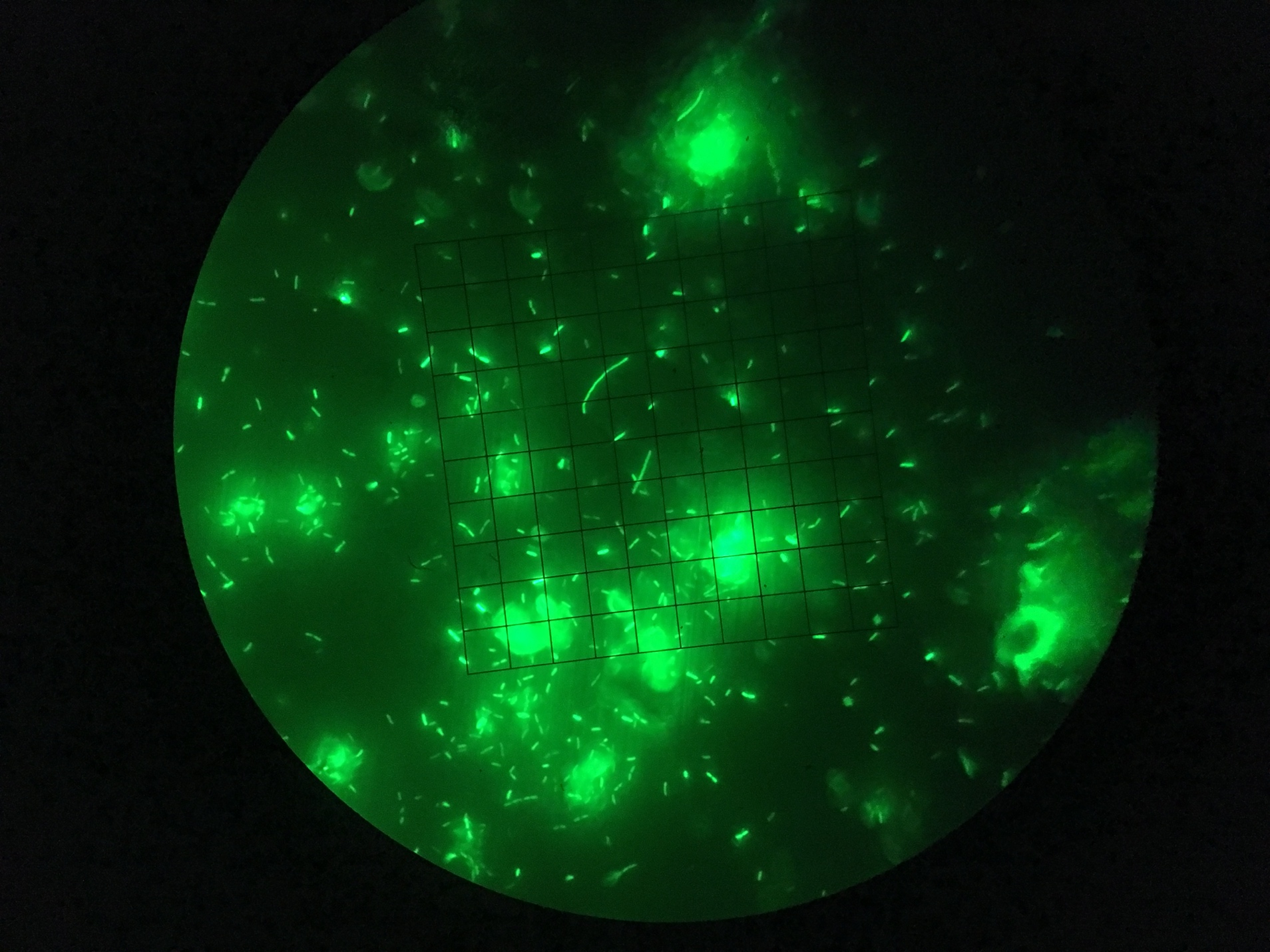


**Supplementary Figure S1** Fluorescence micrograph of an active culture of *Shewanella oneidensis* mixed in a 1:1 ratio with wFGD slurry

**Supplementary Table S1** Cell counts of wFGD slurries.

| Site | Slide | Field | Live Cell Count | Dead Cell Count |
| --- | --- | --- | --- | --- |
| S | 1 | 1 | 0 | 2 |
| S | 1 | 2 | 1 | 0 |
| S | 1 | 3 | 1 | 0 |
| S | 1 | 4 | 4 | 3 |
| S | 1 | 5 | 1 | 2 |
| S | 2 | 1 | 1 | 5 |
| S | 2 | 2 | 6 | 2 |
| S | 2 | 3 | 2 | 3 |
| S | 2 | 4 | 5 | 8 |
| S | 2 | 5 | 2 | 1 |
| S | 3 | 1 | 3 | 0 |
| S | 3 | 2 | 1 | 1 |
| S | 3 | 3 | 3 | 0 |
| S | 3 | 4 | 3 | 2 |
| S | 3 | 5 | 6 | 0 |
| M | 1 | 1 | 3 | 2 |
| M | 1 | 2 | 3 | 4 |
| M | 1 | 3 | 3 | 3 |
| M | 1 | 4 | 4 | 3 |
| M | 1 | 5 | 0 | 2 |
| M | 2 | 1 | 1 | 1 |
| M | 2 | 2 | 0 | 2 |
| M | 2 | 3 | 1 | 4 |
| M | 2 | 4 | 2 | 5 |
| M | 2 | 5 | 4 | 4 |
| M | 3 | 1 | 1 | 0 |
| M | 3 | 2 | 0 | 1 |
| M | 3 | 3 | 3 | 0 |
| M | 3 | 4 | 1 | 2 |
| M | 3 | 5 | 0 | 5 |
| P | 1 | 1 | 7 | 2 |
| P | 1 | 2 | 8 | 0 |
| P | 1 | 3 | 12 | 4 |
| P | 1 | 4 | 7 | 0 |
| P | 1 | 5 | 6 | 1 |
| P | 2 | 1 | 1 | 1 |
| P | 2 | 2 | 5 | 1 |
| P | 2 | 3 | 4 | 1 |
| P | 2 | 4 | 4 | 0 |
| P | 2 | 5 | 3 | 0 |
| P | 3 | 1 | 13 | 2 |
| P | 3 | 2 | 8 | 1 |
| P | 3 | 3 | 7 | 5 |
| P | 3 | 4 | 8 | 1 |
| P | 3 | 5 | 15 | 7 |
| P | 4 | 1 | 10 | 2 |
| P | 4 | 2 | 8 | 1 |
| P | 4 | 3 | 4 | 1 |
| P | 4 | 4 | 5 | 0 |
| P | 4 | 5 | 5 | 0 |
| P | 5 | 1 | 21 | 1 |
| P | 5 | 2 | 11 | 1 |
| P | 5 | 3 | 7 | 0 |
| P | 5 | 4 | 6 | 0 |
| P | 5 | 5 | 8 | 2 |

**Supplementary Table S2. Estimates of microbial abundances and diversity of wFGD systems.**

| **Site** | **No. of Sequences** | **No. of unique OTUs** | **Shannon Index** |
| --- | --- | --- | --- |
| wFGD S Slurry | 102,895 | 2,991 | 6.02 |
| W FGD M Slurry | 140,147 | 2,728 | 4.49 |
| wFGD P Slurry | 86,851 | 1,665 | 4.54 |
| wFGD S Source Water | 135,615 | 4,123 | 6.80 |
| W FGD P Source Water | 134,310 | 4,092 | 6.08 |

**Supplementary Table S3.** Metadata from metatranscriptomic sequencing.

Upload: bp Count 697,898,504 bp

Upload: Sequences Count 4,083,031

Upload: Mean Sequence Length 171 ± 58 bp

Upload: Mean GC percent 47 ± 9 %

Artificial Duplicate Reads: Sequence Count 0

Post QC: bp Count 694,910,829 bp

Post QC: Sequences Count 4,083,031

Post QC: Mean Sequence Length 170 ± 59 bp

Post QC: Mean GC percent 47 ± 10 %

Processed: Predicted Protein Features 74,406

Processed: Predicted rRNA Features 679

Alignment: Identified Protein Features 6,989

Alignment: Identified rRNA Features 158

Annotation: Identified Functional Categories undefined


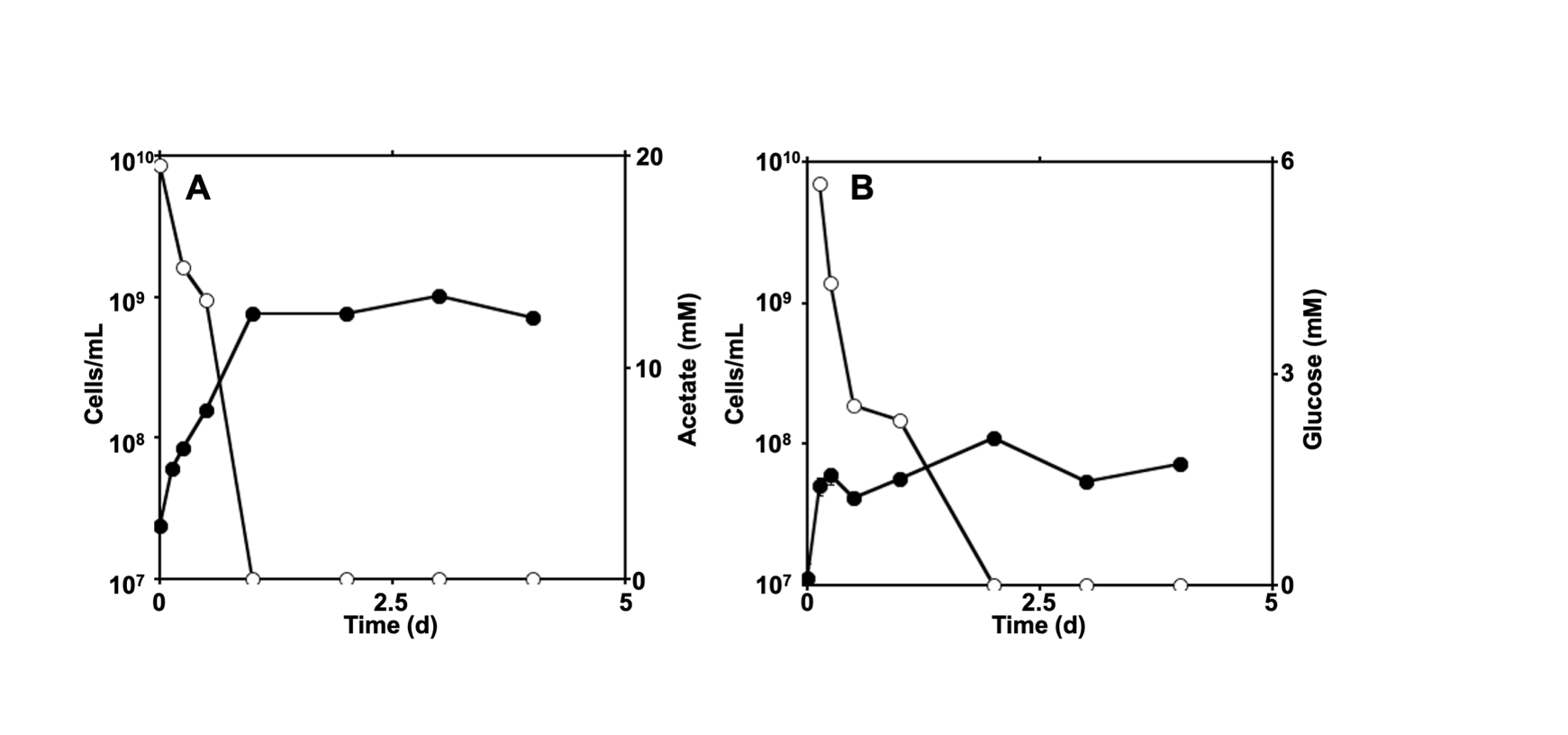


**Supplementary Figure S2** Cell abundance (⚫) and electron donor concentration (⭘) in (A) acetate-oxidizing and (B) glucose oxidizing enrichment cultures from wFGDs
